# Genetic insights into the non-indigenous copepod *Pseudodiaptomus marinus* Sato, 1913 (Calanoida: Pseudodiaptomidae) in the Mediterranean Sea

**DOI:** 10.7717/peerj.21752

**Published:** 2026-10-20

**Authors:** Iole Di Capua, Roberta Piredda, Alberto Amato, Elisa Camatti, Ylenia Carotenuto, Stefano Corona, Rosa D’Angiolo, Andrea Marcucci, Alenka Goruppi, Valentina Tirelli, Giacomo Zagami, Marco Uttieri

**Affiliations:** 1 Stazione Zoologica Anton Dohrn, Villa Comunale 1, Naples, Italy; 2 NBFC, National Biodiversity Future Center, Palermo, Italy; 3 Laboratoire de Physiologie Cellulaire et Végétale, Université Grenoble-Alpes CEA CNRS INRAE IRIG-CEA, Grenoble, France; 4 Institute of Marine Science (ISMAR), National Research Council (CNR), Venice, VE, Italy; 5 National Institute of Oceanography and Applied Geophysics—OGS, Trieste, Italy; 6 Department of Veterinary Sciences, University of Messina, Messina, Italy; 7 CONISMA–Consorzio Nazionale Interuniversitario per le Scienze del Mare, Rome, Italy

**Keywords:** Genetic diversity, COI, ITS, Non-indigenous species, Molecular taxonomy, Pseudodiaptomidae, *Pseudodiaptomus marinus*

## Abstract

The calanoid copepod *Pseudodiaptomus marinus* Sato, 1913, native to the Indo-Pacific region, has rapidly expanded its range in European waters since its first detection in the Adriatic Sea in 2007, raising questions about its introduction pathways and population dynamics. In this study, we investigated the genetic diversity and phylogeography of *P. marinus* in the Mediterranean Sea, focusing on four Italian coastal areas, using a dual-marker approach based on the mitochondrial cytochrome oxidase subunit I (COI mtDNA) and the internal transcribed spacer of the ribosomal cistron (ITS) genes. Molecular data allowed the reconstruction of the phylogenetic relationships within the Pseudodiaptomidae family and the characterization of the species *P. marinus* across geographically distant areas. COI analyses revealed three main Pseudodiaptomidae clades corresponding to freshwater, brackish, and marine species, while all *P. marinus* sequences clustered together, showing no evidence of cryptic species. Haplotype networks indicate repeated human-mediated introductions, with Italian populations connected among themselves and to other introduced areas, though higher haplotype diversity was observed in putative native regions. ITS analyses confirmed the absence of clear biogeographic structuring among Italian populations, suggesting that some ITS sequences from Korean populations, within or near the presumed native range of *P. marinus*, may require further verification, although the observed divergence could also reflect native-range genetic diversity or distinct lineages within a broader species complex. Overall, our findings provide an overview of the genetic diversity of *P. marinus* across different Mediterranean areas, offering new insights into its characterization and suggesting possible introduction pathways in European waters. These findings underscore the value of integrated molecular approaches in supporting and enhancing monitoring programs for non-indigenous species, while also improving our ability to anticipate their potential spread under future environmental changes scenarios.

## Introduction

The Mediterranean Sea is a renowned hotspot of both endemic biodiversity and biological invasions (Tsirintanis et al., 2022). Several drivers have been hypothesized to be at play, including changes in sea surface temperature, increased propagule pressure, and augmented research efforts (as reviewed in Zenetos et al., 2022). Over an 11-year time window (2010–2021), the number of established non-indigenous species (NIS) has increased by 41%, scoring a total of ∼1,000 validated introductions (Zenetos et al., 2022). Estimating the actual number of introduced species is made difficult by numerous factors, including the lack of taxonomic expertise and the presence of species complexes (Bonanno & Orlando-Bonaca, 2019; Katsanevakis & Moustakas, 2018). The impact of introduced species in the Mediterranean basin is still largely unknown (Bonanno & Orlando-Bonaca, 2019). The impacts of Mediterranean NIS are context-dependent, with both positive and negative effects reported according to the ecological target descriptor considered, namely biodiversity, ecosystem services, and human health (Tsirintanis et al., 2022).

In terms of Mediterranean subregions, as identified by the European Environment Agency (2020), the Western Mediterranean Sea is the most invaded, with Italy, France and Spain scoring the highest records (Tsiamis et al., 2019). Given its particular position, Italian seas belong to three different subregions: the Ligurian and Tyrrhenian Sea to the Western Mediterranean Sea (MWE); the Ionian Sea, together with the southern and eastern coasts of Sicily, to the Ionian Sea and the Central Mediterranean Sea (MIC); and the Adriatic Sea to the homonymous subregion (MAD) (European Environment Agency, 2020). The trend in Italian introductions matches that of the Mediterranean one: a total of 111 species was censused by Occhipinti-Ambrogi (2002), rising first to 165 (Occhipinti-Ambrogi et al., 2011) and then to 265 in less than two decades (Servello et al., 2019). This last figure is even more staggering considering that it equals nearly 25% of the introductions at the Mediterranean level (Zenetos et al., 2022). The Italian NIS are equally distributed among the MWE, MIC and MAD, and a large proportion of them are considered established (Servello et al., 2019). Crustaceans represent one of the most relevant groups among Italian marine NIS, with decapods being the dominant component, while planktonic copepods currently account for a smaller but increasing fraction of introduced taxa (Di Capua & Boxshall, 2008; Di Capua & Mazzocchi, 2021). Recent Mediterranean assessments further confirm Italy as one of the Mediterranean countries with the highest numbers of recorded marine NIS and highlight the continuing spread of crustacean invaders across different subregions (Galanidi et al., 2023).

Following its first record in the Adriatic Sea in 2007 (De Olazabal & Tirelli, 2011), the calanoid copepod *Pseudodiaptomus marinus* Sato, 1913, native to the Inland Sea of Japan (Ohtsuka et al., 2018), has increasingly spread its distribution in European seas and neighbouring waters (Sabia et al., 2015; Uttieri et al., 2020; Uttieri et al., 2023). *P. marinus* is widely present in Italian waters, occurring in the coastal sectors of the Ligurian, Tyrrhenian, Ionian and Adriatic Sea (Corona et al., 2026; Uttieri et al., 2023). This species can inhabit a wide range of environments, from freshwater to hypersaline, due to specific biological and physiological traits (*e.g.*, Sabia et al., 2017; Sabia et al., 2014; Svetlichny et al., 2019).

Molecular tools are increasingly used not only to identify individuals at the species level, in the framework of the so-called “integrative taxonomy” (Dayrat, 2005; Will, Mishler & Wheeler, 2005), but also to gain insights into the range of expansion and the source of introduction of NIS (*e.g.*, Bonanno & Orlando-Bonaca, 2019 and references therein). A multi-marker approach is often suggested as a proper approach to reduce the risk of species misidentifications (*e.g.*, Varrella et al., 2025; Zhang et al., 2018), and it can also provide information on the distribution and the differentiation of NIS (Ghabooli et al., 2013). Among the various alternative genes, the mitochondrial cytochrome oxidase subunit I (COI mtDNA) is one of the most commonly used for copepods, allowing reliable identification down to the species level (*e.g.*, Bucklin, Steinke & Blanco-Bercial, 2011; Laakmann, Blanco-Bercial & Cornils, 2020). As reviewed in Bucklin, Steinke & Blanco-Bercial (2011), COI offers numerous advantages compared to other mitochondrial genes, and it has been successfully used as a diagnostic tool for cryptic species and for unveiling hidden diversity (*e.g.*, Blanco-Bercial et al., 2014; Bucklin et al., 2010; Di Capua et al., 2022; Di Capua et al., 2023). The internal transcribed spacer of the ribosomal cistron (ITS) is another gene that can be considered a suitable alternative to COI (Bucklin, Steinke & Blanco-Bercial, 2011). It is frequently used in phylogenetic and phylogeographic studies and is valued for its high variability and sequencing efficiency (Rocha et al., 2019). ITS has been successfully applied in copepods and has also demonstrated its utility in species from other orders (*e.g.*, Chen & Hare, 2008; Di Capua et al., 2017; Jeon et al., 2018; Miller, Bradford-Grieve & Jillett, 1999; Sabia et al., 2017). Additionally, analyses of ITS2 secondary structure and Compensatory Base Changes (CBCs) are used to refine species delimitation and assess the potential for interbreeding among genetically divergent populations.

By integrating mitochondrial and nuclear markers, our findings reveal patterns of genetic diversity among geographically distant populations of *P. marinus*, providing new insights into its spread, characterization, and possible introduction pathways in Mediterranean and European waters.

## Materials & Methods

### Study areas

The specimens of *P. marinus* were collected at four sites along the Italian coasts (Fig. 1): the Gulf of Trieste (GoT; Fig. 1A) and the Venice Lagoon (VL; Fig. 1B) in the Northern Adriatic Sea (MAD); Torre del Greco (TdG; Fig. 1C), a coastal site in the Gulf of Naples (GoN) in the Central Tyrrhenian Sea (MWE); and Lake Faro (LF; Fig. 1D), located at the northeastern tip of Sicily, at the boundary between the Central Tyrrhenian Sea (MWE) and the Ionian Sea (MIC).

**Figure 1:**
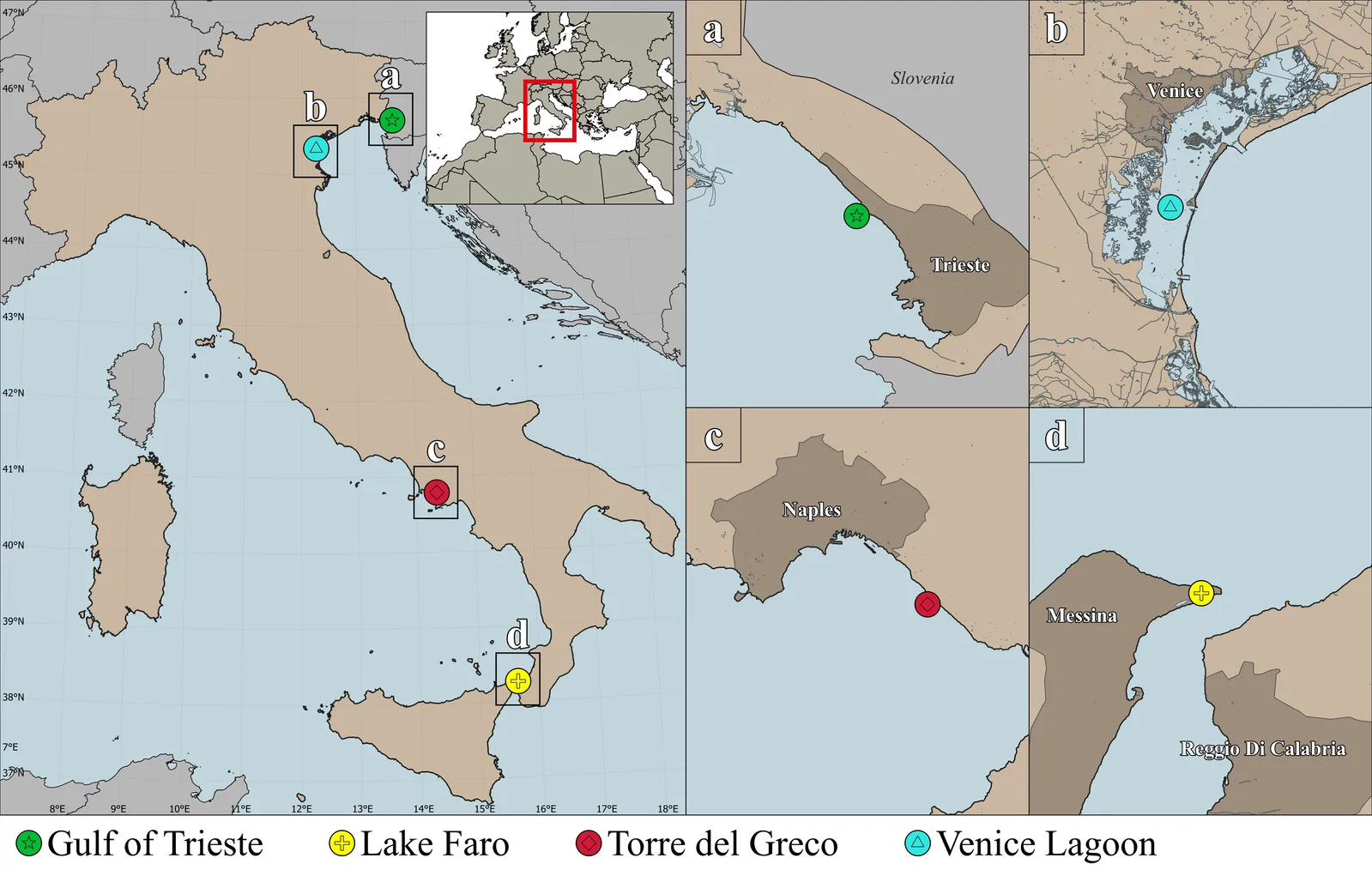
Study area and sampling site along Italian coasts. (A) Gulf of Trieste (GoT); (B) Venice Lagoon (VL); (C) Torre del Greco (TdG) in the Gulf of Naples (GoN) and (D) Lake Faro (LF).

The GoT, in the northernmost part of the Adriatic Sea, is a semi-enclosed coastal area of >600 km^2^ and maximum depth of 26 m (Olivotti, Faganelli & Malej, 1986) (Fig. 1A). The individuals of *P. marinus* were recorded for the first time in this basin in an artificial canal near the port of Monfalcone (Italy) in May 2009 (De Olazabal & Tirelli, 2011). In the following years, observations of this NIS increased, at several sites in the central area of the GoT, in the ports of Trieste and Monfalcone, at the coastal Long-Term Ecological Research (LTER) Golfo di Trieste site and in the Marano and Grado Lagoon (Uttieri et al., 2020; Uttieri et al., 2023).

The VL is a large Mediterranean lagoon covering ∼500 km^2^ along the northwest coast of the Adriatic Sea (Fig. 1B). The depth of the lagoon is very shallow, averaging about 1.3 m, but it reaches over 10 m in the deepest channels (Ghezzo et al., 2011). The lagoon is surrounded by urban and industrial zones, and activities such as fishing, mariculture, and tourism are highly developed (Solidoro et al., 2010). The combination of simultaneous stressors determines high habitat heterogeneity (Camatti et al., 2023). The first record of *P. marinus* in the port of Venice dates to May 2014 (Vidjak et al., 2019), followed by additional records both in the lagoon and in the Gulf of Venice between 2014 and 2017 (Uttieri et al., 2020).

The GoN is a coastal sector of the Central Tyrrhenian Sea subject to intense anthropogenic pressure (Cianelli et al., 2012) (Fig. 1C). The first record of *P. marinus* dates back to July 2011, through eDNA barcoding (V4-18S rRNA; Di Capua et al., 2021). This NIS has been recurrently found as a rare species at the coastal LTER MareChiara site (https://deims.org/0b87459a-da3c-45af-a3e1-cb1508519411) (Mazzocchi et al., 2023; Uttieri et al., 2020; Uttieri et al., 2023), but occasionally also in offshore waters (Sabia et al., 2015), in the southeasternmost part of the GoN (TdG, Torre Annunziata and Sarno River mouth; Uttieri et al., 2023), and in the Gulf of Pozzuoli (a sub-basin of the GoN; Margiotta et al., 2020).

The LF is part of the Laguna di Capo Peloro natural reserve (Bertolino et al., 2022; Somma et al., 2024) (Fig. 1D). It has a quasi-permanent anoxic layer below 10 m, owing to its unique bathymetric contour and meromictic regime (Saccà, Guglielmo & Bruni, 2008; Somma et al., 2024). Over the years, an increasing number of NIS has been recorded in the LF, including protozoans, sponges, polychaetes, and copepods (as reviewed in Bertolino et al., 2022). *P. marinus* has been recorded in the LF since October 2008 (Sabia et al., 2014), becoming the fourth copepod species in terms of abundance (Pansera et al., 2014).

### *Pseudodiaptomus marinus* sampling and morphological identification

*P. marinus* specimens were sorted from mesozooplankton samples that were collected using standard vertical nets with a 200 µm mesh and preserved in 95% ethanol. In the Gulf of Trieste (GoT; LTER site Golfo di Trieste, https://deims.org/96969205-cfdf-41d8-979f-ff881ea8dc8b: 45.69911N, 13.70765E), samples were collected by vertical hauls of a WP2 net (57 cm mouth Ø) from near-bottom (18 m depth) to the surface. In the Venice Lagoon (VL; LTER Venice Lagoon site, https://deims.org/96969205-cfdf-41d8-979f-ff881ea8dc8b: 45.337N, 12.2747E), zooplankton was sampled using horizontal tows with an Apstein net equipped with a flowmeter to measure the filtered water volume, at a depth of 0.5 m. At TdG (40.7864N, 14.3517E) samples were collected by vertical hauls using a WP2 plankton net from 90 m depth to the surface, while in LF (38.269N, 15.637E), vertical tows with a WP2 net were conducted within the oxygenated layer (15 m to the surface).

*P. marinus* is currently the only representative of the genus reported in European waters, and its identification is supported by established taxonomic keys (Zagami, Bonanzinga & Pansera, 2023). Diagnostic features include the separation between the head and first thoracic segment, the presence of pointed posterolateral spines on the prosome, and the characteristic female P5 morphology.

Adult females were identified under stereomicroscopes at each sampling site (GoT: Olympus SZX16 and Leica M165C; VL: Zeiss Stemi 2000-C; TdG: Leica M165C; LF: Wild MDG-17). In addition, all female specimens were morphometrically examined, and high-definition images were acquired before DNA extraction.

### Molecular analyses

The extraction, purification, and amplification of *P. marinus* DNA followed the modified cetyltrimethylammonium bromide (CTAB) protocol discussed in Di Capua et al. (2022). COI was amplified using the Folmer et al. (1994) primers pairs (forward LCO1490: 5′-GGTCAACAAATCATAAAGATATTGG-3′; reverse HCO2198: 5′-TAAACTTCAGGGTGACCAAAAAATCA-3′), while ITS region was amplified using the forward ITS1 (5′-TCCGTAGGTGAACCTGCGG-3′) and reverse ITS4 (5′-TCCTCCGCTTATTGATATGC-3′) primers designed in White et al. (1990).

BioEdit Sequence Alignment Editor 7.0.5.3 (Hall, 1999) was used to obtain consensus sequences from forward and reverse, while mothur v.1.44.3 (Schloss et al., 2009) collapsed identical sequences. Molecular identifications for all generated sequences were confirmed by the similarity approach against the National Center for Biotechnology Information (NCBI) (Altschul et al., 1997) and (only for COI) also against the Barcode of Life Data Systems database (BOLD) (Meiklejohn, Damaso & Robertson, 2019; Ratnasingham & Hebert, 2007). For the COI dataset, we also used a reference sequence (NCBI accession number: MZ710190; Di Capua et al., 2022) obtained in the GoN (LTER site MareChiara, https://deims.org/0b87459a-da3c-45af-a3e1-cb1508519411, depth ca. 75 m). A phylogenetic tree was built using the ten COI sequences generated in this study and the 65 sequences downloaded from NCBI and BOLD databases (Table S1). *Pleuromamma xiphias* (Giesbrecht, 1889) (NCBI accession number: JN574428) was used as the outgroup. In detail, multialignment was generated using MAFFT v. 7 (Katoh, Rozewicki & Yamada, 2019; Kuraku et al., 2013) and manually checked using SeaView v.4.0 (Gouy, Guindon & Gascuel, 2010). A maximum-likelihood (ML) tree was inferred with IQ-TREE 2 software (Minh et al., 2020) using TIM+F+I+G4 substitution model, selected as best-fit model by ModelFinder within IQ-TREE (Kalyaanamoorthy et al., 2017), and 500 replicate bootstraps. The tree was visualized in iTOL4 (Letunic & Bork, 2019). Moreover, intraspecies population relationships were explored, producing phylogenetic networks generated both for COI and ITS markers using all the sequences available for *P. marinus*. Haplotype lists were generated with DnaSp v. 6 (Rozas et al., 2017), then Median-Joining (MJ) haplotype networks were inferred with Network 10 (Bandelt, Forster & Röhl, 1999) using default parameters.

The secondary structure of the ITS2 region was predicted using mfold software (Zuker, 2003) and visualised in the PseudoViewer 3 Web Application (Byun & Han, 2009). The margins of each region were identified by secondary structure predictions using the ViennaRNA Web Service (Gruber, Bernhart & Lorenz, 2015). In particular, the ITS2 secondary structure prediction and CBC/Hemi-Compensatory Base Change (HCBC) search analyses were carried out (Coleman, 2003). The absence of CBCs in helix III, the most conserved ITS2 region, was verified to ensure that individuals belonged to the same biological species (Coleman, 2000). The *P. marinus* ITS sequences used in Sabia et al. (2017) were aligned with the sequences produced in the present study using MAFFT v. 7 (Katoh & Standley, 2013), and a ML tree was built following the same workflow described above for COI.

## Results

A total of ten COI and ten ITS reference sequences of *P. marinus* were successfully amplified and sequenced in this study (Table 1).

**Table 1:** New COI and ITS consensus sequences obtained for the different sites investigated.

**Sites**	**Individual code**	**COI (bp)**	**NCBI accession no**	**ITS (bp)**	**NCBI accession no**
Gulf of Trieste (GoT)	Pmar_Trieste_Z1	576	PZ441184	no	no
	Pmar_Trieste_Z2	576	PZ441185	no	no
	Pmar_Trieste_Z13	no	no	667	PZ441404
Venice Lagoon (VL)	Pmar_Venezia_Z1	no	no	620	PZ441405
	Pmar_Venezia_Z2	576	PZ441183	no	no
	Pmar_Venezia_Z3	no	no	694	PZ441406
	Pmar_Venezia_Z4	576	PZ441186	418	PZ441408
	Pmar_Venezia_Z6	576	PZ441187	615	PZ441409
Gulf of Naples –Torre del Greco (TdG)	Pmar_Torre_N19	576	PZ441178	695	PZ441402
	Pmar_Torre_N20	576	PZ441179	687	PZ441400
Lake Faro (LF)	Pmar_Faro_Z7	576	PZ441180	651	PZ441401
	Pmar_Faro_Z8	576	PZ441181	566	PZ441403
	Pmar_Faro_Z10	576	PZ441182	696	PZ441407

The COI phylogenetic tree consisted of a total of 75 sequences including all the references for Pseudodiaptomidae genera and *P. marinus* (Fig. 2 and Table S1). The tree included 21 different species grouped in three main clades following their adaptation to different habitats. Indeed, the first clade included ten species mainly inhabiting freshwater systems belonging to the genera *Skistodiaptomus* and *Acanthodiaptomus*, and the species *Pseudodiaptomus marshi* Wright S., 1936. The second clade grouped *Calanipedia aquaedulcis* Krichagin, 1873 and nine *Pseudodiaptomus* brackish water species. The third clade comprised marine/brackish species belonging to the genus *Pseudodiaptomus*: *P. marinus* sequences from various geographic regions, including our newly obtained sequences, as well as *P. ishigakiensis* Nishida, 1985 and *P. nihonkaiensis* Hirakawa, 1983 (Fig. 2).

**Figure 2:**
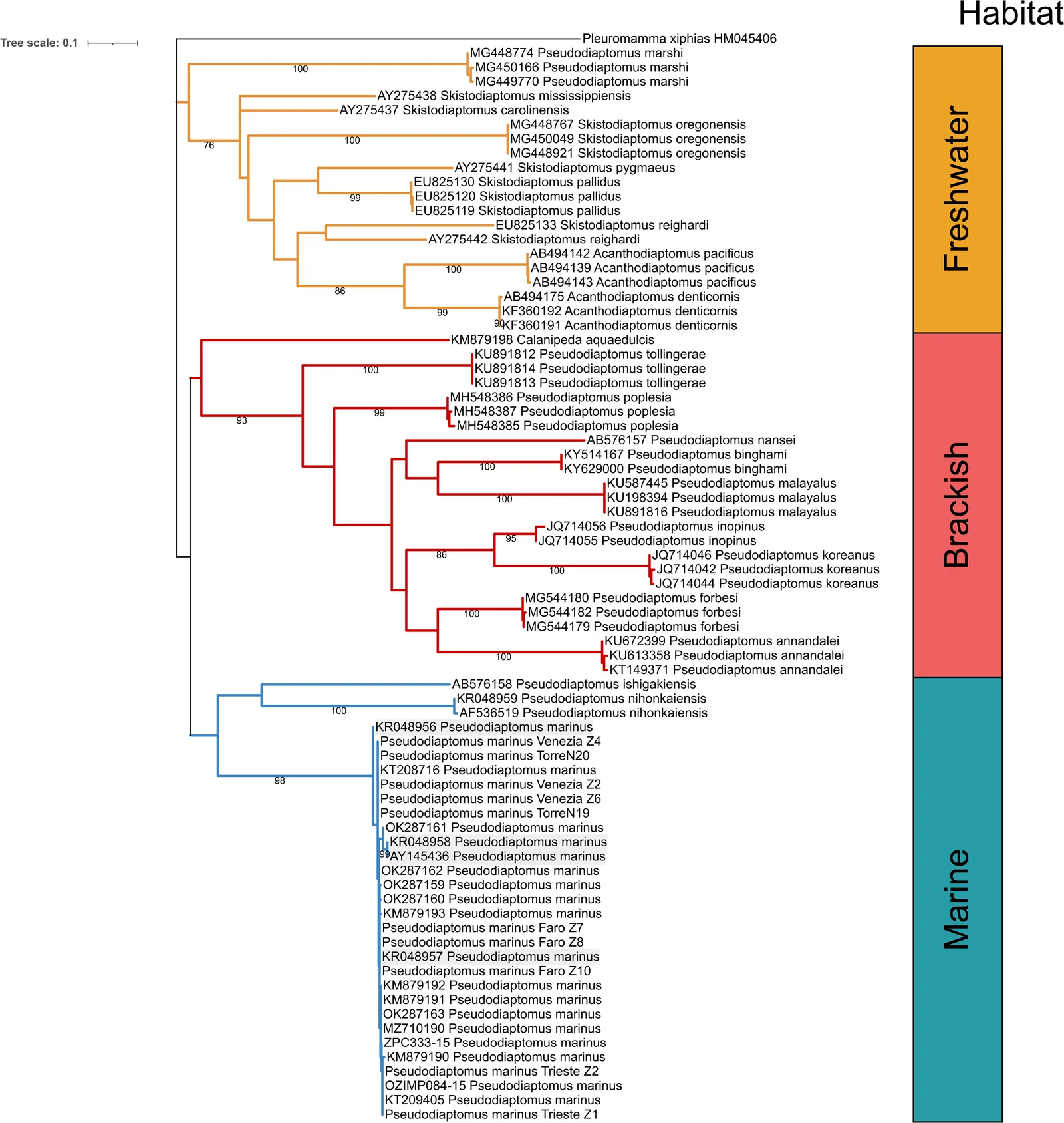
Pseudodiaptomidae phylogeny. Maximum Likelihood tree of 75 COI sequences including the ten sequences generated in this study and 65 sequences downloaded. *Pleuromamma xiphias* (JN574428) was used as outgroup. Bootstrap values ¿ 70% are indicated in the tree. *Pseudododiaptomus marinus* sequences from Korea were highlighted with grey background in label.

The haplotype networks generated using all the sequences available for *P. marinus* consisted of 28 and 14 sequences for COI and ITS regions, respectively (Fig. 3; Tables S2, S3). For both networks, the references downloaded included sequences generated from Korean areas that were assumed to represent the first genetic signature of the native populations of *P. marinus* (Northwest Pacific Ocean) (Tables S2, S3). Both networks highlighted the lack of a clear biogeographic pattern within *P. marinus* populations (Fig. 3). The COI network (Fig. 3) identified 13 haplotypes, eight of which included one sequence only (Tables S2, S3). Of the four references from Korea, two collapsed in a distinct haplotype (H_7- AY145436 and KR048958), while one was separate in the H_8 (KR048956). The fourth Korean sequence (KR048957) was shared with three individuals from LF (Faro_Z7_COI, Faro_Z8_COI and Faro_Z10_COI) in the H_2 haplotype (Fig. 3). This latter was part of a reticulate structure with other haplotypes from different geographic regions where *P. marinus* has been introduced over time (Mediterranean Sea, Eastern Atlantic Ocean, Eastern Pacific Ocean), without a clear phylogeographic pattern (Fig. 3; Tables S2, S3). Sequences from the GoT were grouped in the H_3 haplotype with sequences from California and the North Sea (Helgoland), while sequences from the VL and TdG were grouped in the H_1 haplotype with those from the North Sea (Helgoland). Finally, the H_5 haplotype included a previous sequence from the GoN (Di Capua et al., 2022) and the sequences from the Marmara Sea (Black Sea) and Bilbao (Iberian Coast).

**Figure 3:**
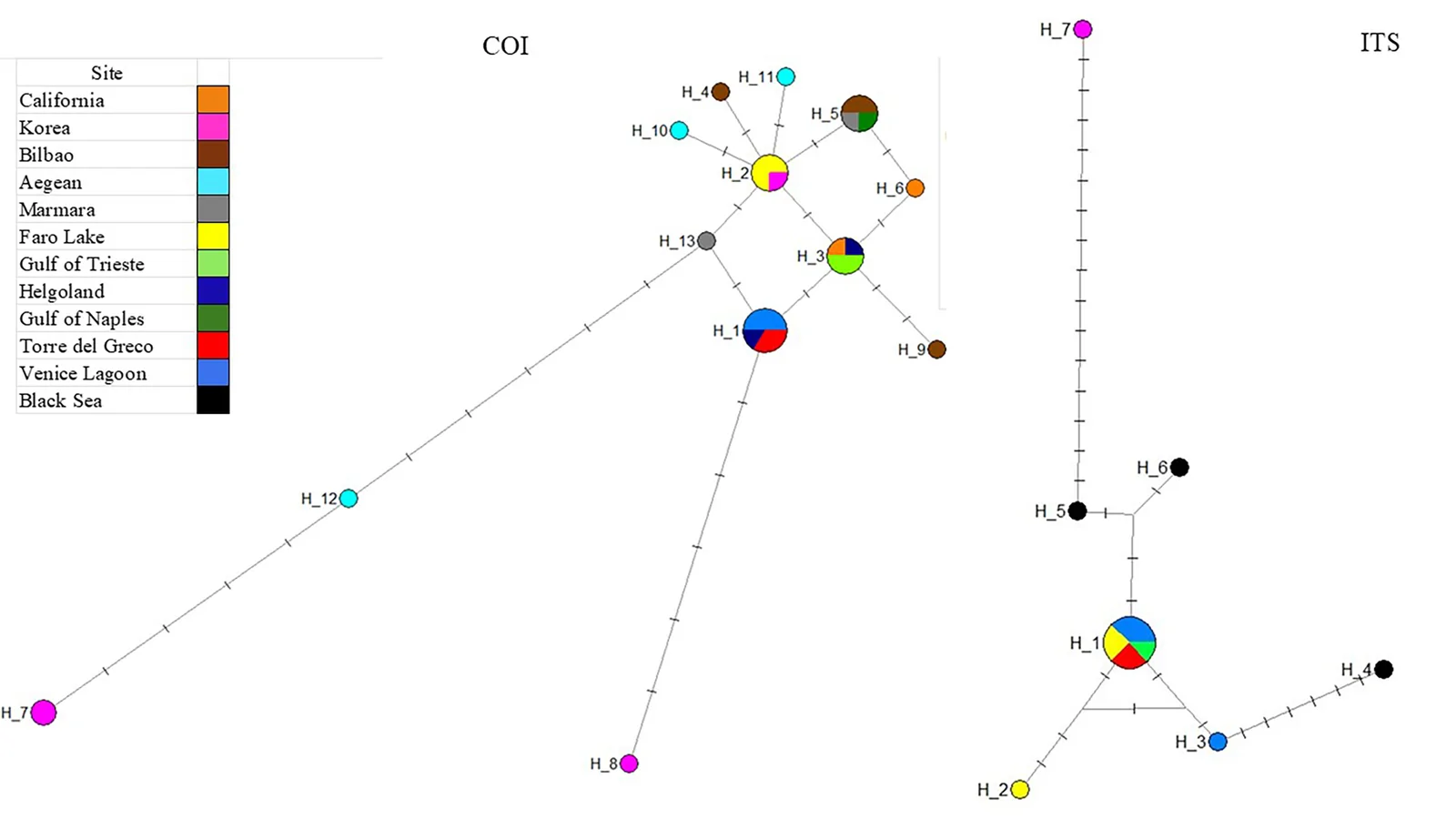
Haplotype networks generated for COI (left) and ITS (right) molecular regions using the sequences of *Pseudododiaptomus marinus* from Italian waters (this study) and all the sequences available from other geographical areas.

ITS sequences produced in the present work were ca. 600 bp long and spanned the ITS1, the 5.8S and the ITS2 including a short portion of 18S and 28S at the 5′ and 3′ ends, respectively. The ITS sequence Faro_Z7_ITS resulted identical to KT808252, the first molecular record of *P. marinus* from the Mediterranean Sea (LF; Sabia et al., 2017). For this reason, only Faro_Z7_ITS was used in the further analyses. The ITS network identified seven haplotypes (Fig. 3). The only ITS sequence available from Korea formed a distinct haplotype (H_7- AY496261), which was clearly separate from the others. All sequences obtained in this study collapsed in the same haplotype (H_1), except for one sequence from LF (H_2) and one from VL (H_3) showing distinct haplotypes. Interestingly, the Black Sea sequences corresponded to three haplotypes (H_4-6), one of which (H_4) clearly separated from the other two. As for the COI, the ITS2 network also revealed no clear biogeographic pattern (Fig. 3).

To enhance the resolution of the genetic relationships among different populations, ITS2 secondary structure prediction and CBC search analyses were carried out. The ITS2 secondary structure predictions revealed a 185 bp long region (identified from the pairing of the 5.8S with the 28S), composed of the four canonical helices with the III being the longest (Fig. 4). The presence of the pyrimidine-pyrimidine mismatch in helix II validated the correctness of the folding. Using Faro_Z7_ITS2 as the reference sequence (Fig. 4), the analysis of the Italian and Black Sea sequences revealed no CBC in helix III, whereas three CBCs and two HCBCs were detected compared to the Korean reference. Helix I was the most variable among the analysed sequences, while the tip of helix IV presented subtle differences (Fig. S1).

**Figure 4:**
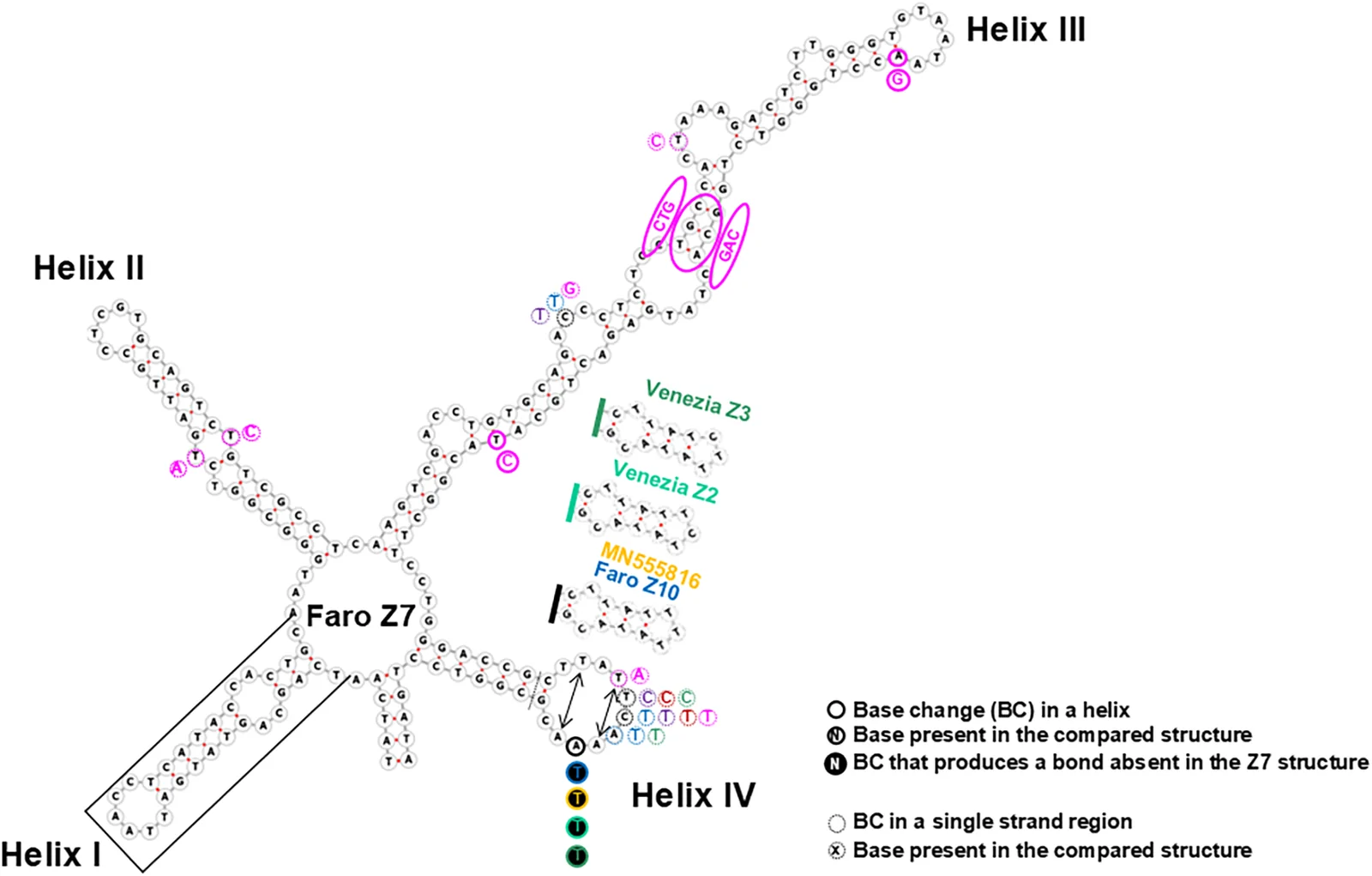
The predicted secondary structures of the ITS2 helices of *P. marinus*. The identified CBCs were shown on the helices.

## Discussion

Zooplankton invasions are reported almost worldwide (Dexter & Bollens, 2020). In marine ecosystems, copepods are particularly efficient colonizers, with widely documented intercontinental introductions (*e.g.*, Cordell et al., 2008; Ruiz et al., 2011; Villate, Uriarte & Iriarte, 2018; Zagami et al., 2018). Their success is largely attributed to the variety of anthropogenic vectors they can exploit (Choi et al., 2005; Feis et al., 2019; Velasquez et al., 2021; Vidjak et al., 2019). Among calanoid copepods, the genus *Pseudodiaptomus* includes several NIS of global concern, such as *Pseudodiaptomus forbesi* and *Pseudodiaptomus inopinus* (Adams, Bollens & Bishop, 2015; Bollens et al., 2012; Bollens et al., 2014; Cordell & Morrison, 1996; Cordell, Rasmussen & Bollens, 2007).

The dual-marker approach (COI and ITS) used in this study reinforces the taxonomic resolution for *P. marinus*, supporting its reliable molecular identification across the four investigated Italian sites and contributing to a broader set of validated reference sequences for the Mediterranean Sea (Di Capua et al., 2022; Kourkoutmani et al., 2026; Sabia et al., 2017; Tiralongo et al., 2022; Tirelli et al., 2024). Furthermore, the COI and ITS genes, incorporating ITS2 secondary structure and CBCs as additional features, were applied for species delimitation also within Harpacticoida and provided the first molecular data for a harpacticoid species from Türkiye (Bakmaz, Sönmez & Korkmaz, 2025). These findings reinforce the results of the present study and further highlight the need for a comprehensive taxonomic revision of the family, integrating both morphological and molecular approaches to clarify species boundaries and phylogenetic relationships.

The COI phylogenetic tree distinguishes three main Pseudodiaptomidae clades, corresponding to freshwater, brackish, and marine species, in agreement with previous ecological groupings based on ITS2 (Sabia et al., 2017). Overall, our results do not support the existence of cryptic species within *P. marinus* populations sampled across different biogeographic regions: all European and extra-European sequences cluster together, and both COI and ITS haplotype networks show no clear geographic structuring. While some haplotypes are shared across regions, this pattern suggests repeated human-mediated introductions rather than ongoing gene flow.

*P. marinus* COI data support the occurrence of high genetic diversity within the species, particularly in Korean populations, as previously reported by Ohtsuka et al. (2018). However, the limited geographic coverage and incomplete metadata associated with several sequences deposited in public databases currently hamper a robust reconstruction of introduction routes and source populations. The reticulate structure of the COI haplotype network, showing shared or closely related haplotypes among Mediterranean, European Atlantic, Southern North Sea, and Eastern Pacific populations, is consistent with repeated introduction events followed by secondary spread across invaded regions (Uttieri et al., 2020; Uttieri et al., 2023). Such a pattern likely reflects the role of maritime traffic and port connectivity in facilitating dispersal.

Analyses based on the ITS marker, particularly the ITS2 region, confirm the absence of clear biogeographic structuring among Italian *P. marinus* haplotypes, while highlighting higher differentiation in Black Sea populations, consistent with previous findings from Sevastopol Bay (Gubanova et al., 2020). The lack of CBCs in helix III between Italian and Black Sea specimens supports their conspecific status and suggests potential gene flow between these populations. In contrast, the presence of CBCs and HCBCs in sequences attributed to Korean populations (AY496261; Gubanova et al., 2020) suggests a possible misidentifications, emphasizing the importance of an integrated molecular-assisted *α*-taxonomy approach, correct identifications and the need for robust, validated sequence databases (Di Capua et al., 2022; Di Capua et al., 2023; Peters et al., 2025), or indicating a markedly higher divergence among different population. Differences observed in helix I of the ITS2 secondary structure remain difficult to interpret due to limited comparative data for *Pseudodiaptomus*. Further integrative analyses combining multiple species concepts, together with broader sampling across the native distribution range of *P. marinus*, are needed to clarify the taxonomic significance of these patterns and the phylogenetic position of the Korean lineage. Nonetheless, although ITS sequences of *P. marinus* are still relatively scarce in public repositories, our findings confirm ITS as a powerful gene for species delimitation and detection of cryptic diversity (Li et al., 2024; Liu et al., 2024; Van Wormhoudt et al., 2019). Future studies integrating ITS with other molecular markers will be essential to further elucidate the bioinvasion dynamics and evolutionary trajectories of planktonic species (*e.g.*, Kochanova et al., 2021; Kourkoutmani et al., 2026; Mouth et al., 2024).

## Conclusions

*P. marinus* forms a well-supported genetic clade, with Italian populations showing genetic connections not only among themselves but also with those from other introduced areas, without evident geographic patterns. At the same time, higher haplotype diversity is observed in the presumed native area of origin, supporting the hypothesis of multiple introduction events followed by secondary dispersal in invaded areas. Overall, the present study provides additional insight into the genetic diversity and phylogeography of *P. marinus*, contributing to a better understanding of its invasion pathways. Given the ongoing environmental changes and the increasing spread of NIS, investigating the phylogeography of *P. marinus* and other marine invaders remains essential for understanding the evolutionary and ecological processes that facilitate adaptation, establishment, and persistence in newly colonized environments.

## Supplemental Information

10.7717/peerj.21752/supp-1 Supplemental Information 1 List of COI and ITS sequences used in the manuscript List of COI sequences used to build phylogenetic tree. List of COI sequences used to generate haplotype network. Haplotypes with frequency higher than 1 were indicated in red. List of ITS sequences used to generate haplotype network. Haplotypes with frequency higher than 1 were indicated in red.

10.7717/peerj.21752/supp-2 Supplemental Information 2 Sequences COI

10.7717/peerj.21752/supp-3 Supplemental Information 3 Sequences ITS

10.7717/peerj.21752/supp-4 Supplemental Information 4 CBC search in elix I of the IT2 secondary structure

## Additional Information and Declarations

### DNA Deposition

The following information was supplied regarding the deposition of DNA sequences: The raw sequence data are available at NCBI. The complete list of accessions is available in the Supplemental Files.

### Data Availability

The following information was supplied regarding data availability: The raw measurements are available in the Supplemental Files. The raw sequence data are available at NCBI. The complete list of accessions is available in the Supplemental Files.
